# Thermal Impedance Characterization Using Optical Measurement Assisted by Multi-Physics Simulation for Multi-Chip SiC MOSFET Module

**DOI:** 10.3390/mi11121060

**Published:** 2020-11-30

**Authors:** Min-Ki Kim, Sang Won Yoon

**Affiliations:** Department of Automotive Engineering, Hanyang University, Seoul 04763, Korea; automk@hanyang.ac.kr

**Keywords:** thermal impedance, multi-chip, SiC MOSFET, power module

## Abstract

In this paper, an approach to determine the thermal impedance of a multi-chip silicon carbide (SiC) power module is proposed, by fusing optical measurement and multi-physics simulations. The tested power module consists of four parallel SiC metal-oxide semiconductor field-effect transistors (MOSFETs) and four parallel SiC Schottky barrier diodes. This study mainly relies on junction temperature measurements performed using fiber optic temperature sensors instead of temperature-sensitive electrical parameters (TESPs). However, the fiber optics provide a relatively slow response compared to other available TSEP measurement methods and cannot detect fast responses. Therefore, the region corresponding to undetected signals is estimated via multi-physics simulations of the power module. This method provides a compensated cooling curve. We analyze the thermal resistance using network identification by deconvolution (NID). The estimated thermal resistance is compared to that obtained via a conventional method, and the difference is 3.8%. The proposed fusion method is accurate and reliable and does not require additional circuits or calibrations.

## 1. Introduction

Power modules with high power densities are increasingly in demand for various applications, including electrified vehicles, transportations, and renewable energy systems [1,2,3]. The reliabilities and performances of power modules are strongly dependent on the selection of switching devices. Recently, wide-band-gap devices such as SiC and gallium nitride (GaN) devices have been actively studied because of their potential to overcome the limitations of Si devices [4,5,6]. For high-power applications, SiC metal-oxide semiconductor field-effect transistors (MOSFETs) are commonly used for parallel operations [7,8]. In addition, they are often paired with anti-parallel diodes (Schottky barrier diodes (SBDs)) to avoid potential reliability issues of the body diode [9,10].

It is expected that such parallel SiC power modules would encounter various thermal reliability issues originating from their high power density. Common thermal reliability issues include thermal stress, thermal fatigue, and wire bond lift-off caused by thermal expansion coefficient mismatches and large temperature fluctuations [11,12,13,14,15]. Thus, thermal reliability analysis is crucial for parallel SiC devices. One of the most commonly used parameters is thermal impedance.

The thermal impedance is composed of thermal resistance and thermal capacitance. The thermal impedance can be derived from the junction temperature cooling curve. Thus, for an accurate thermal impedance characterization, device junction temperature monitoring is essential. Junction temperatures are primarily monitored by either measuring temperature-sensitive electrical parameters (TSEPs) or employing additional temperature sensors [16,17,18,19,20,21,22,23,24]. Commercial equipment can automatically generate thermal impedance curves via TSEP measurement [25]. Various studies have been carried out on measurements or estimations of the device junction temperature of a power module, as summarized in Table 1.

TSEPs are highly effective indicators that can be employed for the rapid monitoring of the junction temperature [16,17,18,19,20]. The body diode of the MOSFET is used to estimate the junction temperature via its forward voltage change [16,17]. The gate threshold voltage (*V*_gs_) is also an effective parameter because its reduction is linear with an increase or decrease in the junction temperature [17]. Numerous studies related to drain-source saturation voltage (*V*_On_) have been carried out. This requires specific *V*_On_ measurement circuits. *V*_On_ can be used to estimate the real-time junction temperature after calibrating *V_O_*_n_ according to the junction temperature [18,19]. In addition, the switching time delay linearly changes with the device junction temperature [19,20]. The switching method shows substantial potential; however, it can be used to measure *T*_j_ only after a switching event. Thus, it is challenging to continuously monitor the temperature changes.

It is also feasible to employ additional temperature sensors in *T*_j_ monitoring. One approach is to integrate a temperature sensor into a power module, whereas another is to employ external equipment. An SiC MOSFET also has an embedded temperature-sensing resistor or diode [21,22]. This monolithic approach is advantageous in terms of directly and accurately measuring *T*_j_. However, the fabrication complexity and cost of such devices is high. Parallel devices require additional sensor-connection wires, which complicates the module design. Miniature temperature sensors are another potential candidate. In addition, negative-temperature-coefficient sensors or piezoelectric thermal-stress sensors [23] have been reported. However, such sensors are not intended to individually monitor the temperature of each device, and thus, further verification is required for multi-chip applications.

Temperature-sensing equipment includes thermocouples, IR cameras, and fiber optic temperature sensors. The thermocouple has the advantages of direct measurement and low price. However, the thermocouple has a slow response and its contact may lead to an additional thermal dissipation path. Thus, it is usually used to verify the thermal resistance with regard to insulation problems. The IR camera provides the temperature distribution in addition to the junction temperature. However, for precise measurement, it is necessary to remove silicone gels filling the power module. A black powder spray is commonly required to avoid reflection problems. Fiber optic temperature sensors represent another option to precisely detect the junction temperature of a target device and its temperature change [24]. However, fiber optics are relatively slow with respect to TSEPs.

As explained above, the currently available *T*_j_ sensing methods have several limitations. This study proposes a new method for fusing *T*_j_ sensing and its estimation. The primary method uses fiber optics because of the high precision and electrical insulation. The optics cannot detect fast responses because of their low speed. The undetected regions are compensated via an estimation based on multi-physics simulations of the power module. The analysis consists of three steps: (1) electrical analysis, (2) steady-state thermal analysis, and (3) transient thermal analysis. After the transient curve is derived using the two methods (fiber optic measurement and simulation-based estimation), the thermal impedance is extracted using network identification by deconvolution (NID), which is a typical thermal impedance extraction method [26,27]. This method yields the thermal resistance, which is compared to *R*_th_ calibrated using a traditional thermal resistance measurement method. The concept of the proposed method is explained in Section 2. Further, Section 3 describes the experimental results obtained based on the fiber optic and conventional methods and multi-physics simulation results. Section 4 presents the validation of the extracted thermal impedance in comparison to the conventional result. The conclusions are presented in Section 5.

## 2. Motivation and Proposed Approach

**Figure 1:**
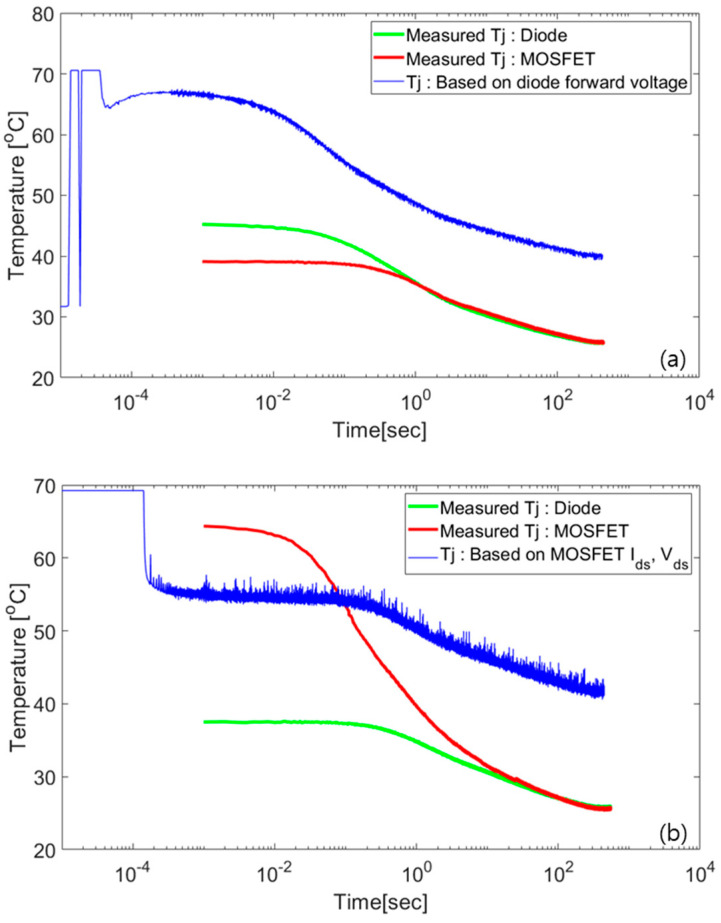
Junction temperature curve during device cooling after heating by a load current of 100 A. Measured junction temperatures of a silicon carbide (SiC) metal-oxide semiconductor field-effect transistors (MOSFET) and SiC diode, compared to (**a**) a temperature-sensitive electrical parameters (TSEP) of the diode forward voltage (*V*_f_) and (**b**) TSEPs of the MOSFET (drain–source current (*I*_ds_) and voltage (*V*_ds_)).

Figure 1 compares the junction temperatures measured by fiber optics and derived from TSEPs. The green and red lines are the *T*_j_ values measured by the fiber optics for a SiC diode and MOSFET, respectively. During the *T*_j_ measurement, either the MOSFET or diode is cooled after applying and removing the load current of 100 A. This process simultaneously heats the MOSFET body diode and its anti-parallel diode and changes their electrical parameters. The forward voltage (*V*_f_) of the diode is monitored as its TSEP and plotted by the blue line in Figure 1a. The TSEP of the MOSFET is derived from the relationship between the drain–source current (*I*_ds_) and voltage (*V*_ds_) under low current (below 100mA), which is expressed by the blue line in Figure 1b.

When the junction temperature is measured in the diode mode using the forward voltage, the junction temperature of the MOSFET is not accurately estimated. The current flows simultaneously between the body diode and SBD, which has a smaller resistance. This leads to an unbalanced current flow. In addition, the cooling performance is different owing to the thickness and chip area of each device, which is discussed in detail in the experimental section. The junction temperature is not accurately measured. The saturation temperature is different from the fiber optic measured temperature, as shown in Figure 1a.

For the measurement of *V*_on_ with *V*_gs_ = 15 V, the measured voltage is unstable in this power module. As shown in Figure 1b, the temperature cooling curve exhibits noise during the cooling. The use of this parameter is challenging in estimating *T*_j_ in multi-chip applications; thus, complex circuits are required.

Contrary to the temperature curve of the TSEP, the temperature measured by the fiber optics is stable and saturated to the ambient temperature (cold plate). The TSEP (*V*_f_ or *V*_ds_) is also saturated, but not to room temperature. Therefore, we set the junction temperature measured by the fiber optics as the actual temperature. However, the temperature measured between 0 and 1 ms (undetected region) is unreliable because the sampling frequency of the fiber optic data acquisition (DAQ) is 1 kHz.

**Figure 2:**
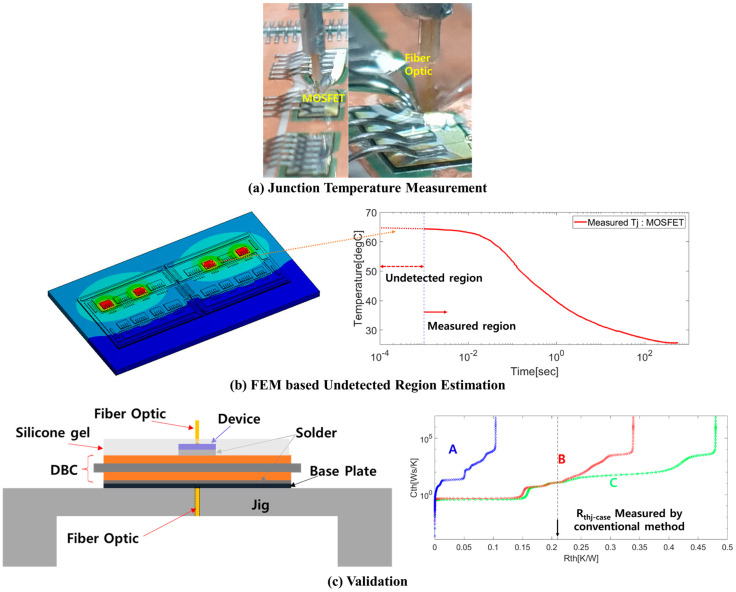
(**a**) Image of the fiber optics on the SiC MOSFET. (**b**) Conceptual image of the undetected region compensated for by the FEM. (**c**) Schematic of the conventional measurement for validation. The graph in (**c**) shows the structure functions obtained by the TSEP and proposed methods (A: structure function based on the TSEP; B: structure function based on the proposed method with thermal grease; C: structure function based on the proposed method without thermal grease).

In this study, the two methods are fused to address the measurement accuracy and slow response issues. The procedure is illustrated in Figure 2. First, we measure the junction temperature via the fiber optics. The temperature measured by the fiber optics is the actual temperature of the SiC MOSFET. Second, the multi-physics finite-element method (FEM) is applied to compensate for the undetected region for the equivalent response time of the TSEP. Third, the thermal resistance is extracted based on the compensated curve and validation is carried out using the conventional method.

## 3. Experiment and Estimation by FEM Simulations

### 3.1. Junction Temperature Measurement

**Figure 3:**
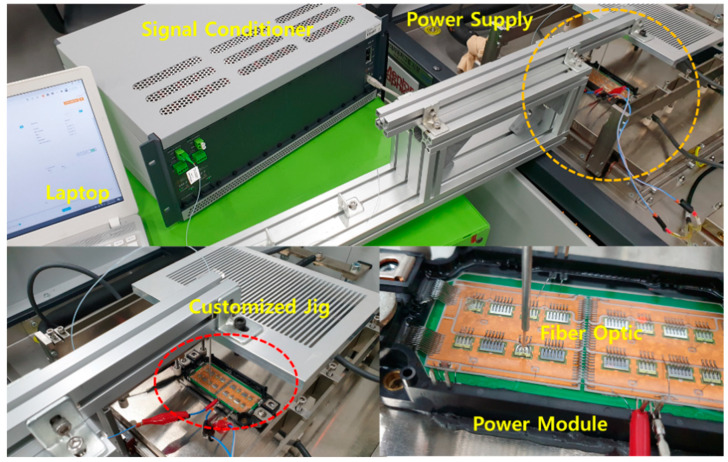
Experimental setup: fiber optic sensor, signal conditioner, power supply, and power module.

The SiC MOSFET power module (SPX300GB120C2S6-2, Semipowerex) is used to validate the proposed method. The module is a 1200 V 300 A half-bridge inverter with four parallel SiC MOSFETs and four parallel SBDs. The direct bonded copper (DBC) is composed of copper and alumina substrate. To heat the SiC MOSFET, the load current is applied using a power source (Mentor Graphics). The power source has a function to measure the drain-source voltage. The power dissipation of the SiC MOSFET is calculated using the measured current load and drain-source voltage. The junction temperature is monitored by an fiber optic sensor (manufactured by OPSENS) with a resolution of 0.05 °C and temperature measurement range of −40 to 250 °C. The fiber optic sensor has low nonlinearity and electrical insulation, and thus is suitable for power device applications. In addition, the junction temperature can be measured even with the gels on the power module. The temperature distribution in the devices can be accurately measured through multi-point sensing. The flexible temperature sensor wire is guided by a customized aluminum fixture with an aluminum tube guide. The sensor tip is touched on the SiC MOSFET to measure the junction temperature. The measured signal is recorded by the signal conditioner, which provides a sampling rate of 1 kHz. The measurement system setup is shown in Figure 3, and its specifications and equipment used are listed in Table 2.

The measurement of the junction temperature cooling curve is carried out according to the following test sequence. When the setup is established as shown in Figure 3, a load current of 120 A is applied by the power source to heat the SiC MOSFET with *V*_gs_ = 15 V. After applying the load current, the MOSFETs are heated until the junction temperature is sufficiently saturated. The saturation criterion is that d*T_j_*/d*t* should reach 0.05 °C/s. The power dissipation is calculated by measuring *V*_ds_ (grease on: 1.08 V; without grease: 1.19 V) and *I*_ds_ (with grease, without grease, 120 A) using a power source. After the power is turned off, the junction temperature is measured until the MOSFET is cooled. The cooling is continued to ambient temperature (initial temperature of the cold plate). The measured temperature is recorded and monitored in real time using a signal conditioner (DAQ). The measurement results will be described in Section 4.3.

### 3.2. Finite Element Method (FEM) Simulation

To compensate for the undetected region, a multi-physics simulation is carried out with modeling based on the module specification sheet. ANSYS, a commercial FEM software package, is used for the multi-physics simulations. An electrical–thermal multi-physics analysis in three steps is carried out. Using the ANSYS Q3D 3D extractor, the volumetric power loss is calculated for the specific power loss of the wires. In the second step, device power dissipation including the generated volumetric heat arising on wires from the first step simulation is imported to perform steady-state thermal analysis. In step 3, the saturated temperature in the steady-state thermal simulation is applied to the transient thermal simulation as the initial value. Through these steps, a cooling curve is obtained.

**Figure 4:**
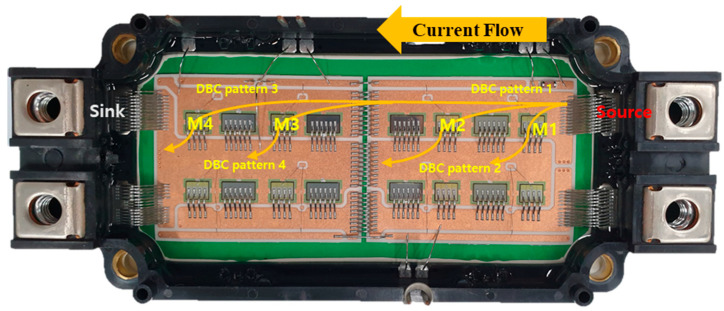
Current flow in power module for ANSYS Q3D simulation: current flow.

The material properties used in the electrical analysis (wire: aluminum, DBC top: copper) are obtained from a library in ANSYS Q3D. The boundary condition is configured for current flow (120 A) from the source to the sink, as shown in Figure 4. The volumetric power loss of the wire bonding on each device to the copper pattern (M1,M2 to DBC pattern 2 and M3,M4 to DBC pattern4) and copper pattern to copper pattern (DBC pattern 1 to 3, DBC pattern 2 to 4) where current flows in Figure 6 is calculated. The total volumetric power loss on the wire bonding is 1.55 W. These simulation results are the initial values of the steady-state thermal analysis (step 2).

**Figure 5:**
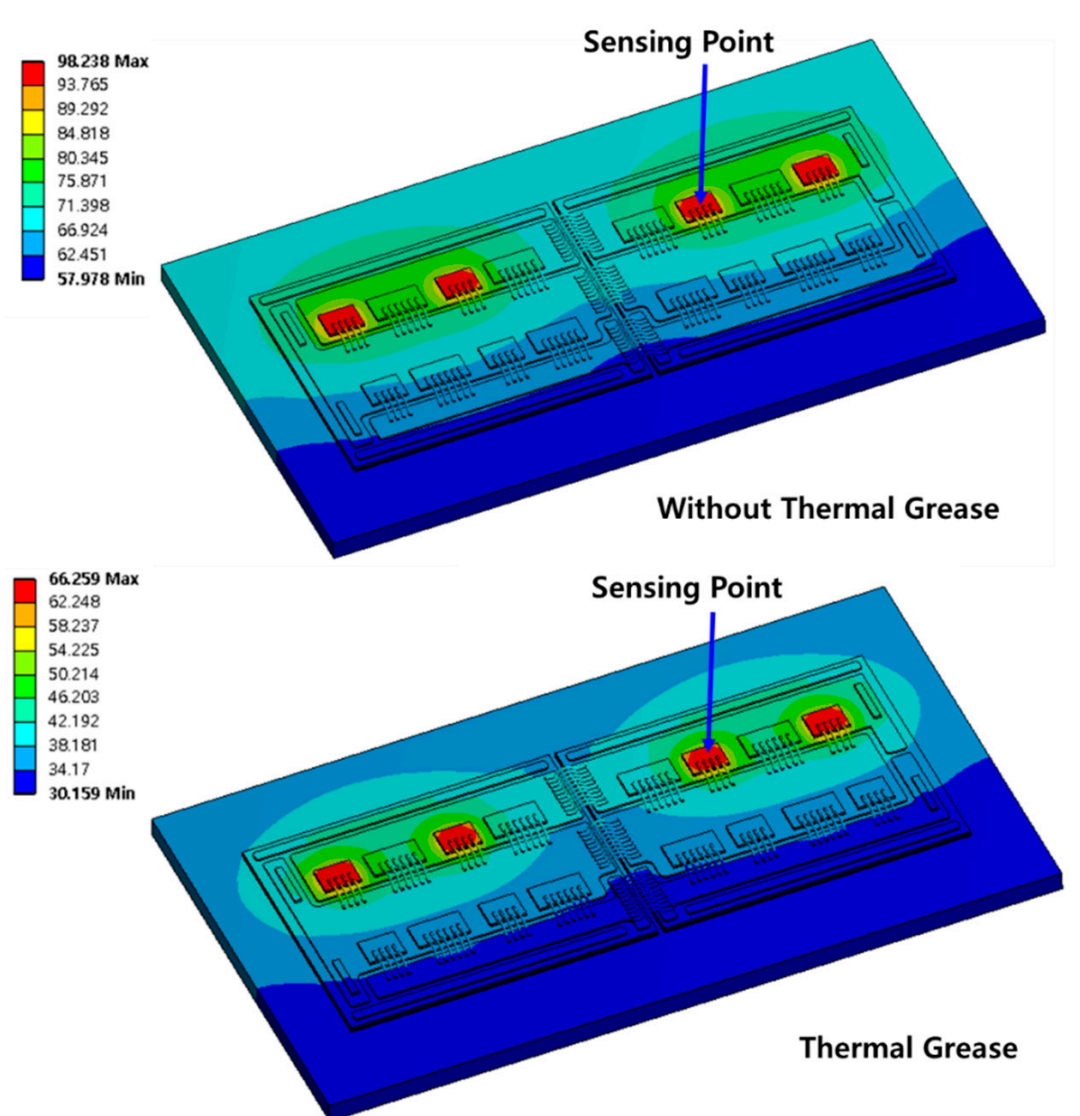
Thermal simulation results (with and without thermal grease).

The material properties of the power module used in the steady-state thermal analysis (step 2) are shown in Table 3. The mesh of each component is generated differently. The device, wire, solder, and DBC top copper are generated with a fine mesh. The other component mesh is relatively coarse to reduce the computation time. For the heating source, the results of step 1 are set in wires as the initial values. The measured heat source (total power loss: 129.6 W (with grease), 142.8 W (without grease)) is applied to the four parallel MOSFETs. The steady-state thermal simulation results are shown in Figure 5. Figure 5 shows the temperature distributions obtained with and without thermal grease, respectively. The maximum temperatures are 66.3 and 98.2 °C, respectively. The temperatures of the sensing point are 66.1 and 97.5 °C, respectively. The simulation results have errors about 1%. They are tuned to match the saturated temperatures corresponding to the experiment and FEM simulation.

A thermal transient simulation is carried out in step 3. The material properties are listed in Table 3. The initial value of the transient simulation is used for the initial temperature before the cooling. The thermal transient analysis is carried out with the heat transfer coefficient corresponding to the bottom of the power module case. Heating source is not applied, except for the initial temperature distribution in step 2. These simulation results are then employed to compensate for the undetected region, as described in Section 4.1.

## 4. Thermal Impedance Extraction and Validation

### 4.1. Cooling Curve Compensation

**Figure 6:**
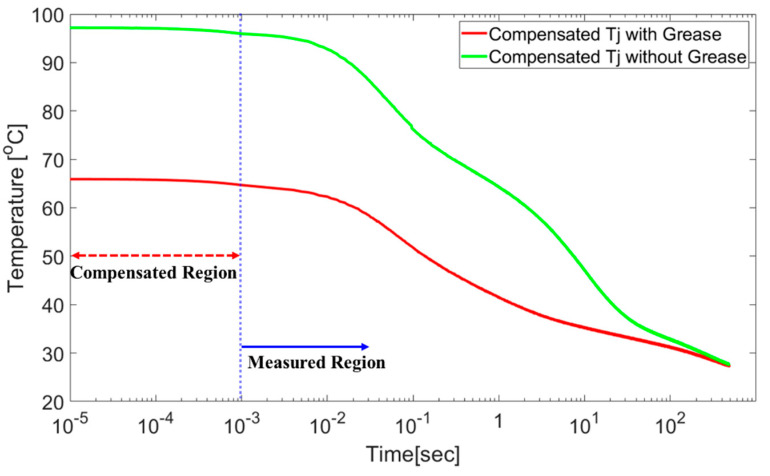
Compensated temperature profile, obtained by multi-physics simulation.

Figure 6 compares the junction temperatures measured by the fiber optics with and without thermal grease, as described in Section 3.1. The green and red lines are the *T*_j_ values measured by the fiber optics for the SiC MOSFET. The blue dotted line indicates and distinguishes the measured and undetected regions. The compensated region is the compensated junction temperature obtained using the multi-physics simulation results presented in Section 3.2. The thermal transient simulation data was filled to the measured data. It is fitted to the equivalent response time with the TSEP. After the compensated data are interpolated, as shown in Figure 6, the thermal resistance is extracted using NID (Section 4.3).

### 4.2. Conventional Method (Reference)

The thermal resistance can be calculated using Equation (1) based on the measured power dissipation and temperature difference of junction to case

(1) $$R_{th}=\frac{T_{j}-T_{case}}{Power\;dissipation}$$

**Figure 7:**
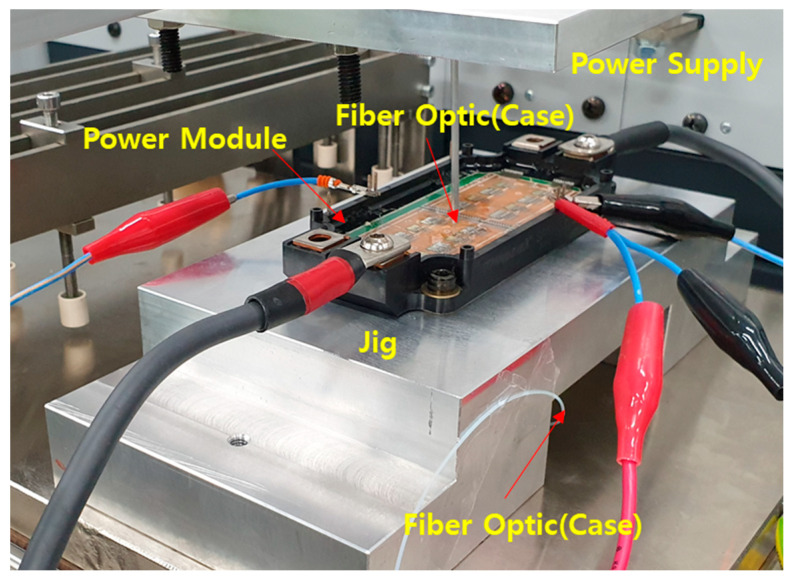
Experimental setup for the conventional method.

The thermal resistance can be obtained by dividing the difference between the device junction temperature and case temperature on the bottom of the aluminum silicon carbide (AlSiC) base plate by the power dissipation of the MOSFETs. The experimental setup with AQG-324 standard [31] is shown in Figure 7. The junction temperature measurement setup is the same as that in Section 3 with an added aluminum jig with a hole at the bottom. The MOSFETs are heated with a load current of 100 A until the difference between *T*_j_ and *T*_case_ is saturated.

**Figure 8:**
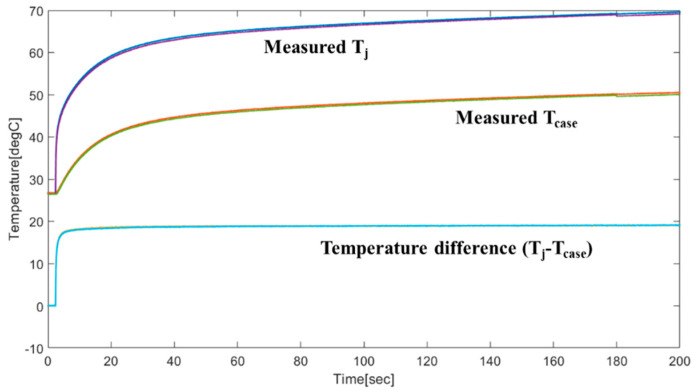
Measured (twice) temperature data: junction temperature, case temperature, and their difference.

The results of repeated experiments are shown in Figure 8. The measured power dissipation is 89 W, while the thermal resistance is calculated to be 0.211 K/W.

### 4.3. Comparison Thermal Resistance

The cumulative structure function is composed of the accumulated thermal resistance and thermal capacitance. In the cumulative structure function, the X axis represents the accumulated thermal resistance (*R*_th_), while the Y axis represents the accumulated thermal capacitance (*C*_th_). These parameters start from the heating point (device junction) and cumulate to the partial thermal resistance and capacitance for the heat flow path.

**Figure 9:**
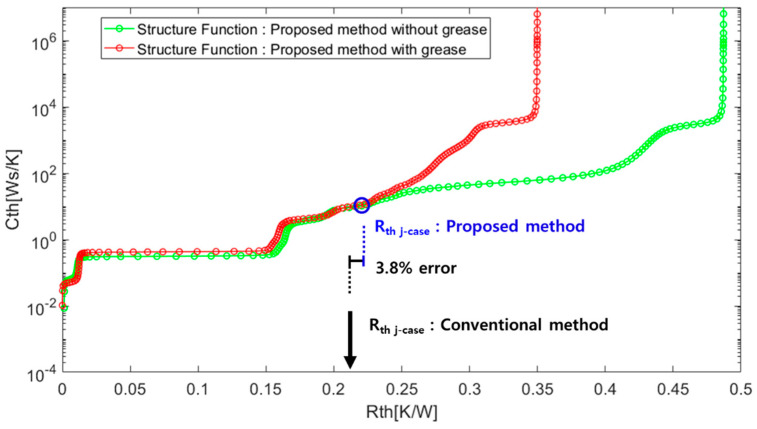
Extracted thermal structure function showing the thermal resistance and capacitance (with and without thermal grease). The thermal resistance measured by the conventional method is marked by the black point.

The cumulative structure function is calculated using a commercial software provided from the T3ster Master from Mentor Graphics. The compensated temperature cooling curve in Figure 6 is imported to the software and analyzed to extract the structure function of the proposed method, as displayed in Figure 9. The extracted junction-to-case thermal resistance (*R*_th j-case_: 0.219 [K/W]) is from the device junction to the base plate. The red line is the structure function extracted based on the temperature curve measured with thermal grease, while the green line is that without thermal grease. The blue circle where the separation starts corresponds to *R*_th j-case_ the junction to the case. A comparison of the proposed and conventional methods shows an error of 3.8%.

## 5. Conclusions

In this paper, we proposed a thermal impedance characterization method based on optical measurements assisted by a multi-physics simulation for a multi-chip SiC MOSFET power module. Fiber optics was used to accurately measure the junction temperature cooling curve. The region that was not detected owing to the relatively slow response of the fiber optic sensor to the TSEP was estimated via the multi-physics simulation. After compensation by the simulation result, the cooling curve was extracted to obtain the thermal resistance and thermal capacitance via the NID method. The results were validated using the conventional measurement method, and the error was within 3.8%. In addition to the high accuracy, the proposed method has the following advantages: (1) simple measurement without employing complex calibrations, (2) measurement availability without removing the silicone gel of the power module, and (3) not requiring additional circuits or additional device pin structures. Thus, this method could be an attractive solution for measuring the thermal impedance in applications entailing multi-chip SiC power modules with anti-parallel diodes.

## Figures and Tables

**Table 1:** Junction temperature measurements and estimations.

***T*_j_ Measurement or Estimation Method**	**TSEPs**	**Integrated Sensor or External Equipment**
	Forward voltage of body diode [16,17]	Integrated Sensors [21,22,23] (resistive, diode, piezoelectric sensors)
	Gate threshold voltage [17]	Infrared(IR) Camera
	On-state voltage [18,19]	Thermocouple
	Switching time delay, turn-on current transient [19,20]	Fiber optic temperature sensor [24]

**Table 2:** Experiment equipment.

Test Equipment	Specifications and Main Functions
Thermal optics	Temperature range: −40 to 250 °C, resolution: 0.05 °C
Signal conditioner	Sampling rate: 1 kHz
Power source	Power supply current: ~1500 A
Customized jig	Aluminum profile and aluminum tube for guiding the fiber optics

**Table 3:** Material Properties in the finite-element method (FEM) simulation [28,29,30].

Material	Material Properties		
	Density [kg/m^3^]	Thermal Conductivity [W/m·K]	Specific Heat [J/kg·K]
Silicon Carbide	3210	370	750
SAC Solder	7400	50	150
Alumina	3950	25	730
Copper	8850	400	385
AlSiC	3010	200	741
Aluminum	2700	238	880

## References

[1] Zhang H., Tolbert L.M. (2010). Efficiency impact of silicon carbide power electronics for modern wind turbine full scale frequency converter. IEEE Trans. Ind. Electron..

[2] Yoon S.W., Glover M.D., Shiozaki K. (2012). Nickel–tin transient liquid phase bonding toward high-temperature operational power electronics in electrified vehicles. IEEE Trans. Power Electron..

[3] Han T.J., Preston J., Ouwerkerk D. (2013). 6.6 kW on-vehicle charger with a hybrid Si IGBTs and SiC SBDs based booster power module. J. Power Electron..

[4] Cooper J.A., Melloch M.R., Singh R., Agarwal A., Palmour J.W. (2002). Status and prospects for SiC power MOSFETs. IEEE Trans. Electron Devices.

[5] Zhang L., Yuan X., Wu X., Shi C., Zhang J., Zhang Y. (2018). Performance evaluation of high-power SiC MOSFET modules in comparison to Si IGBT modules. IEEE Trans. Power Electron..

[6] Mantooth H.A., Glover M.D., Shepherd P. (2014). Wide bandgap technologies and their implications on miniaturizing power electronic systems. IEEE J. Emerg. Sel. Top. Power Electron..

[7] Fabre J., Ladoux P. (2015). Parallel connection of 1200-V/100-A SiC-MOSFET half-bridge modules. IEEE Trans. Ind. Appl..

[8] Imaizumi M., Miura N. (2014). Characteristics of 600 1200, and 3300 V planar SiC-MOSFETs for energy conversion applications. IEEE Trans. Electron Devices.

[9] Kang M., Yu S., Xing D., Liu T., Salemi A., Booth K., Zhu S., White M.H., Agarwal A.K. Body Diode Reliability of Commercial SiC Power MOSFETs. Proceedings of the 2019 IEEE 7th Workshop on Wide Bandgap Power Devices and Applications (WiPDA).

[10] Yin S., Liu Y., Liu Y., Tseng K.J., Pou J., Simanjorang R. (2017). Comparison of SiC voltage source inverters using synchronous rectification and freewheeling diode. IEEE Trans. Ind. Electron..

[11] Lhommeau T., Perpiñà X., Martin C., Meuret R., Mermet-Guyennet M., Karama M. (2007). Thermal fatigue effects on the temperature distribution inside IGBT modules for zone engine aeronautical applications. Microelectron. Reliab..

[12] Ceccarelli L., Kotecha R.M., Bahman A.S., Iannuzzo F., Mantooth H. (2019). Mission-profile-based lifetime prediction for a SiC MOSFET power module using a multi-step condition-mapping simulation strategy. IEEE Trans. on Power Electron..

[13] Lee H., Smet V., Tummala R. (2019). A Review of SiC Power Module Packaging Technologies: Challenges, Advances, and Emerging Issues. IEEE J. Emerg. Sel. Top. Power Electron..

[14] Sugiura K., Iwashige T., Tsuruta K., Chen C., Nagao S., Funaki T., Suganuma K. (2019). Reliability evaluation of SiC power module with sintered Ag die attach and stress-relaxation structure. IEEE Trans. Compon. Packag. Manuf. Technol..

[15] Luo H., Iannuzzo F., Baker N., Blaabjerg F., Li W., He X. (2019). Study of current density influence on bond wire degradation rate in SiC MOSFET modules. IEEE J. Emerg. Sel. Top. Power Electron..

[16] Gonzalez J.O., Alatise O., Hu J., Ran L., Mawby P.A. (2016). An investigation of temperature-sensitive electrical parameters for SiC power MOSFETs. IEEE Trans. Power Electron..

[17] Avenas Y., Dupont L., Khatir Z. (2011). Temperature measurement of power semiconductor devices by thermo-sensitive electrical parameters—A review. IEEE Trans. Power Electron..

[18] Degrenne N., Brandelero J., Kawahara C., Mollov S. (2020). A Review on the Application of On-line Von (On-State Voltage) Sensing for Junction Temperature Estimation of Power Semiconductor Modules. Proceedings of the CIPS 2020, 11th International Conference on Integrated Power Electronics Systems.

[19] Kalker S., van der Broeck C.H., De Doncker R.W. (2020). Online Junction-Temperature Sensing of SiC MOSFETs with Minimal Calibration Effort. Proceedings of the PCIM Europe digital days 2020; International Exhibition and Conference for Power Electronics, Intelligent Motion, Renewable Energy and Energy Management.

[20] Niu H., Lorenz R.D. (2015). Sensing power MOSFET junction temperature using gate drive turn-on current transient properties. IEEE Trans. Ind. Appl..

[21] Berthou M., Godignon P., Millan J. (2013). Monolithically integrated temperature sensor in silicon carbide power MOSFETs. IEEE Trans. Power Electron..

[22] Kempiak C., Lindemann A., Idaka S., Thal E. (2020). Comparative Study of Determining Junction Temperature of SiC MOSFETs during Power Cycling Tests by A Tj Sensor and The VSD (T)-Method. Proceedings of the CIPS 2020; 11th International Conference on Integrated Power Electronics Systems.

[23] Kim M.K., Yoon S.W. (2018). Miniature piezoelectric sensor for in-situ temperature monitoring of silicon and silicon carbide power modules operating at high temperature. IEEE Trans. Ind. Appl..

[24] Choi U.M., Blaabjerg F., Jørgensen S. (2017). Power cycling test methods for reliability assessment of power device modules in respect to temperature stress. IEEE Trans. Power Electron..

[25] Mentor Graphics (2013). T3Ster—Thermal Transient Tester—Technical Information.

[26] Szekely V. (1998). Identification of RC networks by deconvolution: Chances and limits. IEEE Trans. Circuits Syst. I Fundam. Theory Appl..

[27] Lasance C.J.M., Poppe A. (2014). Thermal Management for LED Applications.

[28] Hu B., Gonzalez J.O., Ran L., Ren H., Zeng Z., Lai W., Gao B., Alatise O., Lu H., Bailey C. (2017). Failure and reliability analysis of a SiC power module based on stress comparison to a Si device. IEEE Trans. Device Mater. Rel..

[29] Chen C., Luo F., Kang Y. (2017). A review of SiC power module packaging: Layout, material system and integration. Cpss Trans. Power Electron. Appl..

[30] Li J., Castellazzi A., Eleffendi M.A., Gurpinar E., Johnson C.M., Mills L. (2017). A physical RC network model for electrothermal analysis of a multichip SiC power module. IEEE Trans. Power Electron..

[31] (2019). ECPE Guideline AQG 324—Qualification of Power Modules for Use in Power Electronic Converter Units in Motor Vehicles. https://www.ecpe.org/research/working-groups/automotive-aqg-324/.

